# Temporary amnesia from sleep loss: A framework for understanding consequences of sleep deprivation

**DOI:** 10.3389/fnins.2023.1134757

**Published:** 2023-03-30

**Authors:** Paul Whitney, Courtney A. Kurinec, John M. Hinson

**Affiliations:** ^1^Department of Psychology, Washington State University, Pullman, WA, United States; ^2^Sleep and Performance Research Center, Washington State University, Spokane, WA, United States; ^3^Elson S. Floyd College of Medicine, Washington State University, Spokane, WA, United States

**Keywords:** sleep deprivation, amnesia, memory, binding, decision-making

## Abstract

Throughout its modern history, sleep research has been concerned with both the benefits of sleep and the deleterious impact of sleep disruption for cognition, behavior, and performance. When more specifically examining the impact of sleep on memory and learning, however, research has overwhelmingly focused on how sleep following learning facilitates memory, with less attention paid to how lack of sleep prior to learning can disrupt subsequent memory. Although this imbalance in research emphasis is being more frequently addressed by current investigators, there is a need for a more organized approach to examining the effect of sleep deprivation before learning. The present review briefly describes the generally accepted approach to analyzing effects of sleep deprivation on subsequent memory and learning by means of its effects on encoding. Then, we suggest an alternative framework with which to understand sleep loss and memory in terms of temporary amnesia from sleep loss (TASL). The review covers the well-characterized properties of amnesia arising from medial temporal lobe lesions and shows how the pattern of preserved and impaired aspects of memory in amnesia may also be appearing during sleep loss. The view of the TASL framework is that amnesia and the amnesia-like deficits observed during sleep deprivation not only affect memory processes but will also be apparent in cognitive processes that rely on those memory processes, such as decision-making. Adoption of the TASL framework encourages movement away from traditional explanations based on narrowly defined domains of memory functioning, such as encoding, and taking instead a more expansive view of how brain structures that support memory, such as the hippocampus, interact with higher structures, such as the prefrontal cortex, to produce complex cognition and behavioral performance, and how this interaction may be compromised by sleep disruption.

## 1. Introduction

There is a considerable history of interest in the relationship between sleep and memory, but since the seminal studies on sleep and memory (Jenkins and Dallenbach, 1924), most research addressing this relationship has focused on how sleep strengthens memories established during previous waking hours. More recently, there has been increased study of the ability to learn and remember new information while sleep deprived. There are good reasons, both practical and theoretical, to better understand how sleep deprivation may impair or degrade the quality of memory. With epidemic levels of insufficient sleep in modern industrial societies (Chattu et al., 2018), the potential for impaired learning due to lack of sleep is very high in schools and training environments. From a fundamental research perspective, if we are to identify the mechanisms by which sleep deprivation affects cognitive performance in general, memory is a critical domain to understand because what we remember from our experiences affects how we relate to others and how we make choices. Recognizing the much larger body of sleep research on how sleep strengthens previously acquired memories, it is noteworthy that Newbury et al. (2021) found that sleep deprivation after learning produced substantially smaller effects than a night of sleep deprivation before learning new material.

The effects of sleep deprivation before or after learning are typically mapped onto a division of memory processing into three phases: encoding, consolidation, and retrieval. Research on sleep deprivation after learning is most concerned with the role of sleep in memory consolidation. According to the *active systems view* of the consolidation of memory, sleep facilitates the transfer of information from hippocampally dependent processing more broadly to the neocortex where it is integrated with prior knowledge (Takashima et al., 2009; Walker, 2009; Born and Wilhelm, 2012; Rasch and Born, 2013). Research on sleep deprivation before learning analyzes possible impairments in the other phases of memory. The consensus from this research is that sleep deprivation degrades the capacity for encoding new information, and similar to studies of memory consolidation, has focused on the hippocampus and associated structures where sleep loss decreases hippocampal activation and alters its connectivity with other brain regions. Specifically, neuroimaging studies indicate that sleep deprivation decreases connectivity with the prefrontal cortex (PFC), temporal, and parietal lobes, and increases connectivity with alertness networks that include the thalamus (Yeo et al., 2015; Zhao et al., 2019; Chai et al., 2020; Gisabella et al., 2020).

Our purpose here is to provide a framework for better understanding the growing body of evidence on the effects of sleep deprivation before learning. Although there are typically large effects of sleep deprivation on subsequent learning, multiple investigators have noted that there is substantial variation in these effects for reasons that are as yet unknown (Cousins and Fernández, 2019; Vaseghi et al., 2021). We believe that lessons from the history of studies of memory impairment from hippocampal lesions, which produce anterograde amnesia, suggest that viewing sleep deprivation as producing a deficit in “encoding capacity” may be misleading and may obscure important connections between memorial effects of sleep loss and other cognitive processes not typically considered in a memory context.

## 2. Lessons from the study of amnesia

One of the most famous patients in the history of clinical neurology, Henry Molaison, known in the scientific and medical literature by his initials H.M. until after his death, provided the most important case study of profound anterograde amnesia resulting from bilateral resection of the medial temporal lobes (MTL). Study of H.M. had an extraordinary influence on subsequent memory research. This influence, and that of converging studies of other individuals with hippocampal damage, has been widely discussed and we need not provide a comprehensive review here (*cf.*, Scoville and Milner, 1957; Salat et al., 2006; Squire, 2009; Squire and Wixted, 2011). What is important for present purposes is to briefly review changes over time in conceptions of anterograde amnesia, and the role of the hippocampus in memory, as a basis for comparison to current research on sleep deprivation.

Studies of individuals with MTL lesions showed a striking dissociation between an apparent inability to learn new information and intact abilities in short-term memory (STM) and general intellectual functioning. In addition, deficits in memory produced by damage to the hippocampus were not accompanied by deficits in acquiring skills such as mirror drawing and tactile maze learning, or for showing automatic priming of perceptual or conceptual relations among words (Milner et al., 1968; Cohen and Squire, 1980; Levy et al., 2004). Thus, it was concluded that the hippocampus and related structures in the MTL are critical for the transfer of consciously accessible information in short-term memory to more permanent storage in long-term memory (Wickelgren, 1968; Baddeley and Warrington, 1970; Milner et al., 1998; Ranganath and Blumenfeld, 2005). This classic conception of the nature of anterograde amnesia is consistent with the viewpoint that impaired functioning of the hippocampus during sleep deprivation diminishes the capacity to encode new, explicit information. However, as research on amnesia has grown, and with the introduction of functional imaging of the hippocampus during memory tasks, new ideas have supplanted the original conception that the hippocampus functions to transfer information from STM to long-term memory (LTM).

Evidence accumulated from the study of LTM deficits in amnesia clearly demonstrates that the hippocampus supports *relational binding*, i.e., the linking of stimulus elements into integrated representations (Cohen and Eichenbaum, 1993; Ryan et al., 2000), with converging support obtained from animal studies and neuroimaging (Olsen et al., 2012; Bird, 2017; Schwarb et al., 2019). Hippocampally dependent binding occurs across various levels of cognitive processing, from perception to memory (Treisman and Gelade, 1980; Cohen and Eichenbaum, 1993; Yonelinas, 2013). Within the memory domain, binding primarily links stimuli with each other (e.g., associating pairs of words together; Yonelinas et al., 2001), or links stimuli with their relevant contextual information (e.g., remembering when or where an image was viewed; Mitchell and Johnson, 2009). Regardless of what is being bound, successful binding depends on having intact hippocampal functioning, particularly for the creation of complex, high-resolution bindings that help form more precise, differentiated memories (Yonelinas, 2013; Ekstrom and Yonelinas, 2020).

The evidence for relational memory problems in anterograde amnesia comes from a variety of paradigms and stimulus types. For example, MTL damage produces profound deficits in acquiring arbitrary associations of names and pictures (Morrow et al., 2020), binding of objects to the scenes they appear in (Hannula et al., 2015), and the binding of objects to their spatial locations (Horecka et al., 2018). However, it is important to note that the binding problems created by MTL damage will not only be manifest in tests of memory specifically targeting the relationships among stimulus items. Consider the well-known dissociation in which amnesia patients show deficits on explicit memory even as they show unimpaired performance on several tests of implicit memory (Graf et al., 1985). Explicit memories are those that can be consciously and deliberately remembered, such as memories for events and experiences. In contrast, implicit memories are those that are manifest in performance (Squire and Dede, 2015) without awareness, such as habit-based skills or priming, i.e., the influence of stimuli that occurs unconsciously (Schacter, 1987; Squire, 2004; Squire and Dede, 2015). In general, explicit tests of memory will necessarily be dependent on binding of items with their context. Even if asked to simply recognize whether a word was in a study list or not, the context of studying the item in a particular list at a particular time is part of the information that supports a judgment based on recollection of studying the item and, at least for many types of stimuli, whether the word seems familiar (Squire and Wixted, 2011; Bird, 2017). Moreover, the role of the hippocampus in binding also means that its function cuts across the traditional three phases of encoding, consolidation, and retrieval. The associations bound together during encoding are critical to later retrieval because they allow for reinstating earlier hippocampal and cortical activation, even if only partial cues to the original experience are available (Henke, 2010). Reinstating hippocampal-cortical interactions likewise appears to play a role in consolidation (Murty et al., 2017; Cowan et al., 2021).

Although implicit memory is *generally* preserved in amnesia, problems with binding of stimulus elements have also been demonstrated under conditions of implicit memory rather than conscious recollection. Ryan et al. (2000) presented a series of scenes to control subjects and people with amnesia and monitored eye movements as the scenes were viewed. Some scenes were repeated in their original form and other scenes were repeated but with manipulation of the relationships among objects (e.g., shifting the position of one of the objects). Among control subjects, simple repetition reduced visual sampling of the objects in the scene, but manipulated scenes increased viewing time of the changes that were made. These eye movement effects occurred in the absence of conscious awareness of the scene changes. Subjects with amnesia showed the same effect as controls for simple scene repetition, but unlike controls, they showed no effect of changed scenes on viewing times. Thus, people with amnesia, who show severe deficits in conscious recollection, also, in an implicit memory procedure, failed to bind separate objects together in their memory representations.

Additional support for the role of the hippocampus in binding can also be found in studies of amnesia and motor memory. Early studies of the learning of motor procedures through practice, such as mirror drawing and pursuit rotor performance, suggested that motor memory was intact in patients with amnesia (Milner, 1962; Brooks and Baddeley, 1976). While motor skill learning can often occur without hippocampal involvement, particularly in the case of more implicit tasks, subsequent research showed that the hippocampus is critical to such memory when it involves learning higher order motor sequences (Albouy et al., 2013). For example, in the serial reaction time task (SRTT), people use visual cues to anticipate and reproduce a set of corresponding sequential motor responses (Chafee and Ashe, 2007). When the series can be learned from simple pairwise associations among the pattern of cues, amnesic patients do not show learning deficits, but when the pattern depends on more complex higher-order associations among the cues, the hippocampus is needed to bind the motor responses into a sequence. Therefore, if higher order relations must be bound, a process that is hippocampally dependent, amnesic patients show deficits in performance (Curran, 1997; Robertson, 2007).

The classic view that anterograde amnesia represents a failure of encoding information into LTM has been further undermined by studies showing that hippocampal damage disrupts some aspects of STM processing, and not just storage in LTM. For example, when amnesic patients are presented with scenes and are given a recognition memory test after only a few seconds, their memory for which objects are in the scene is typically intact, but memory for item locations within the scenes is very impaired (Olson et al., 2006; Yee et al., 2014). This pattern has been replicated with multiple types of associations among distinct elements including faces and scenes, colors and locations, and colors and numbers (see Olsen et al., 2012 for a review). These results suggest that a critical role of the hippocampus is to bind distinct elements in the focus of attention together into a composite representation that captures the temporal, spatial, and conceptual relationships among the elements (Cohen and Eichenbaum, 1993; Rubin et al., 2017). Results from studies of amnesic patients have received converging support from neuroimaging studies of hippocampal engagement during perceptual processing and working memory tasks (Ranganath and D’Esposito, 2001; Ranganath et al., 2005; Riggs et al., 2009).

Perhaps the most striking departure from the classic view of the hippocampus and amnesia comes from recent research demonstrating that the hippocampus is not limited to functions traditionally designated as being in the memory domain (Olsen et al., 2012; Rubin et al., 2017). Through its role in binding stimulus items together with their context, and through its interaction with other brain areas, particularly the PFC, the hippocampus makes an essential contribution to decision-making, spatial navigation, and some aspects of language use. Amnesia patients once thought to have a circumscribed memory deficit actually show other kinds of deficits related to binding and comparing information (Biderman et al., 2020). For example, performance on the Iowa Gambling Task (IGT), which was originally developed to understand decision-making deficits associated with damage to the ventromedial PFC (Bechara et al., 1994), is impaired in people with MTL damage as well (Bechara et al., 1994; Gutbrod et al., 2006). In the IGT, subjects choose cards from among four decks, and based on the outcomes associated with choices of each deck, they must learn which deck choices are advantageous and which are disadvantageous in the long run. There are multiple possible reasons for poor performance on the IGT, including an insensitivity to future consequences of choices (Bechara et al., 1996). However, in the case of amnesia patients, failure to develop an advantageous choice strategy is likely because the task involves associating gains and losses with their respective decks, i.e., a fundamental binding problem (*cf*., Whitney and Hinson, 2012).

Further evidence that amnesia patients have difficulties in decision-making comes from Bakkour et al. (2019) who reported deficits in value-based decision-making among these patients. Although the patient group performed similarly to controls on a color discrimination task using familiar food items, when asked to compare food items and choose which one they would prefer, a difference between groups emerged. The patient group made choices that were less consistent with their initial evaluation of individual items, while control subjects made value-based comparisons that were highly consistent with initial evaluation of each of the individual items.

In summary, research on amnesia provides compelling evidence that the hippocampus, the functioning of which is known to be strongly affected by sleep deprivation, plays a critical role in LTM through relational binding of stimuli and their context, while also functioning to bind items together in novel associations on timescales operating in perception and working memory. Further, hippocampal binding is needed for performance on several aspects of complex cognition typically considered outside the domain of memory research. We next evaluate whether deficits in these processes associated with anterograde amnesia may underlie sleep deprivation effects on memory and other cognitive processes.

## 3. Temporary amnesia from sleep loss

Based on strong evidence that the hippocampus plays a critical role in relational binding, along with the demonstrated effects of sleep deprivation on hippocampal functioning, we believe it is useful to think of sleep loss effects on memory, and some other aspects of cognition, as representing a case of mild to moderate temporary amnesia. Much like transient global amnesia, which results in cognitive deficits similar to that of hippocampal amnesia but typically resolves with 24 h (Quinette et al., 2003, 2006), the amnesia-like effects of sleep deprivation are expected to resolve without long-term consequences. However, unlike transient global amnesia, the progression of sleep deprivation-induced amnesia is less severe and does not have a sudden and rapid onset. Before addressing the key question of whether such binding problems are manifest under sleep deprivation, we first must acknowledge an important caveat. Sleep deprivation produces some deficits in cognition that are different from problems experienced by patients with MTL lesions. For instance, sleep deprivation disrupts PFC functioning and connectivity with other brain regions (Yoo et al., 2007a), leading to problems directing attentional resources in pursuit of goals (Chee and Tan, 2010) and controlling emotional responses (Stenson et al., 2021). The most noteworthy example is the deficit in vigilant attention produced by sleep deprivation (Lim and Dinges, 2008; Basner and Dinges, 2011; Hudson et al., 2020). Amnesic patients do not typically have problems with vigilant attention, and if a memory task was administered to sleep-deprived subjects in a way that taxes vigilant attention, then lapses of attention could themselves produce a failure to encode stimuli. Nonetheless, in the tests of memory under sleep deprivation discussed below, task pacing and other means of ensuring that stimuli were processed, such as use of orienting tasks, reduce or eliminate lapses of vigilant attention as a potential source of memory deficits.

### 3.1. Binding deficits in explicit and implicit LTM

A comparison of the memory-based deficits observed in amnesia and sleep deprivation is included in Table 1. Like amnesia patients, sleep-deprived subjects show impaired learning and retention of new episodic information, such as lists of words or series of images (Drummond et al., 2000; Walker and Stickgold, 2006; Yoo et al., 2007b). In addition, sleep-deprived subjects show deficits in relational memory, with worse performance on tasks that require binding compared to their baseline performance or their rested counterparts (Harrison and Horne, 2000a; Tempesta et al., 2016; Ratcliff and Van Dongen, 2018; Kurinec et al., 2021). For example, Kurinec et al. (2021) presented subjects, assigned to either a sleep deprived or rested control condition, with a series of words spoken by either a male or female speaker. Compared to their rested counterparts and their own rested baseline, sleep-deprived subjects were worse at recognizing the words presented during study, consistent with previous work showing sleep deprivation impairments in new learning. However, even when sleep-deprived subjects correctly recognized the studied words, they were less able to recognize their associated speaker. Thus, even when sleep-deprived subjects have successfully encoded information, sleep deprivation results in additional impairments to the ability to bind that information with its context.

**Table 1:** Comparison of preserved and impaired memory processes in amnesia and sleep deprivation.

Process	Task	In amnesia	Under sleep deprivation
**Explicit long-term memory**			
Item memory	Encoding of new episodic information (e.g., words or pictures)	Poorer memory for words and pictures than their matched controls (Huppert and Piercy, 1977; Hirst et al., 1986)	Worse memory for words and pictures compared to rested controls or their own rested performance (Drummond et al., 2000; Yoo et al., 2007b)
Relational memory	Associative memory, or memory for the relations among sets of stimuli	Deficit in associative memory for the studied stimuli sets compared to matched controls (Giovanello et al., 2003), regardless of the type of association among the stimuli (Konkel et al., 2008)	Poorer associative and sequential memory for the studied stimuli sets compared to their rested counterparts and/or own performance at rested baseline (Tempesta et al., 2016; Ratcliff and Van Dongen, 2018)
	Source memory, or memory for individual stimuli (items) and the context (source) in which they were originally presented	Worse memory for items and their sources compared to controls (Shimamura and Squire, 1991; Gold et al., 2006)	Worse memory for individual items and their sources compared to rested controls and their own baseline performance (Kurinec et al., 2021)
**Implicit long-term memory**			
Item memory^1^	Unconscious memory for skills	Preserved mirror reading (Cohen and Squire, 1980), mirror drawing, and tactile maze learning (Milner et al., 1968)	Similar encoding of a texture discrimination task as their rested counterparts (McWhirter et al., 2015)
	Priming	Similar conceptual and repetition priming as control subjects (Haist et al., 1991; Levy et al., 2004)	Intact verbal priming compared to their rested baseline (López-Zunini et al., 2014; Casey et al., 2016)
Relational memory	Implicit sequence learning, or learning relationships that occur among series of stimuli	Less able to learn higher-order associations than controls (Curran, 1997)	Poorer learning than their rested counterparts (Heuer et al., 1998; Heuer and Klein, 2003)
**Short-term memory**			
Item memory	Maintaining individual stimuli in the focus of attention	Preserved ability to maintain information in memory (Baddeley and Warrington, 1970; Cave and Squire, 1992)	Preserved working memory scanning (Tucker et al., 2010; Whitney et al., 2015)
Relational memory	Maintaining associations in the focus of attention for short periods of time	Impaired at detecting changes in item-location associations (Yee et al., 2014) and at maintaining object-location associations (Olson et al., 2006)	Poorer performance when spatial positions or color-location pairings must be maintained in memory (Chee and Chuah, 2007; Wee et al., 2013)
**Decision-making**			
Binding independent	Delay discounting, or choosing between smaller immediate rewards or larger, delayed rewards	Show similar discounting functions as matched controls (Kwan et al., 2012, 2013)	Show similar discounting functions as their rested baseline or rested counterparts (Acheson et al., 2007; Libedinsky et al., 2013)
Binding-dependent	Iowa Gambling Task (IGT), which requires subjects to learn to prefer the decks that result in long-term gains	Fail to learn to prefer the advantageous decks over time (Gutbrod et al., 2006; Gupta et al., 2009)	Do not learn to prefer the advantageous decks compared to their rested baseline (Killgore et al., 2006)

1 Although item memory can generally be conceived as a memory representation for a given stimulus, implicit item memory is better thought of as the “functional unit of repetition in a task” (Cohen et al., 1997, p. 150).

There is less of a history of overlapping research paradigms to compare the effects of sleep deprivation and MTL damage on implicit memory. We do know that much like amnesia patients, sleep-deprived subjects show intact priming (López-Zunini et al., 2014; Casey et al., 2016) and preserved performance on some implicit skills (McWhirter et al., 2015). The limited evidence available indicates that sleep-deprived subjects are similarly impaired on implicit memory tasks involving relational memory. For instance, compared to rested controls, sleep-deprived subjects show poorer implicit sequence learning during sleep loss (Heuer et al., 1998; Heuer and Klein, 2003). Implicit sequence learning requires individuals to learn new, higher-order associations, and the acquisition of these relations has been found to activate the MTL in healthy adult subjects, regardless of whether subjects later display awareness of the associations (Schendan et al., 2003). Still, given the limited research on implicit memory and sleep deprivation in general, this pattern of effects must be interpreted cautiously.

There has also been considerable interest in the sleep-related consolidation of motor memories (see King et al., 2017 for a review). Consistent with findings from the amnesia literature indicating a role for hippocampal binding in complex motor sequence learning, the data on sleep-related consolidation show that hippocampal-cortical connections are important when the task is dependent on complex spatial or abstract associations, or explicit memory. However, to our knowledge, there has yet to be a direct test of how sleep deprivation affects simple versus complex motor sequence learning. Adopting tasks used in the amnesia literature that show dissociations in amnesia patients based on the need for binding of relations in implicit or motor learning tasks could provide a strong test of the TASL framework.

### 3.2. Dissociation of STM and LTM

As noted above, the classic dissociation of STM and LTM in performance of amnesic patients is not as clear cut as once believed. Nevertheless, it is certainly the case that hippocampal lesions affect LTM without affecting typical measures of verbal STM, such as digit span. The same is true of subjects under sleep deprivation, as Tucker and colleagues showed that speed of searching verbal STM was unaffected by sleep deprivation (Tucker et al., 2010; García et al., 2021).

The picture is more complex if we look beyond verbal STM, and consider working memory, i.e., functions involved in the storage and manipulation of information in the focus of attention. Working memory can involve manipulation of verbal, semantic, visuospatial, and other types of representations (Baddeley, 1986; Rubin et al., 2017). The precise nature of deficits in working memory associated with amnesia is still being investigated, but as discussed above, there is considerable evidence for the involvement of the hippocampus in working memory processing when binding is needed, particularly when complex spatial relationships among objects must be processed (see Yonelinas, 2013), or when novel rather than familiar stimuli must be maintained (Rose et al., 2012). Consistent with the TASL framework, Chee and Chuah (2007) found a sleep deprivation-induced deficit in performance of a working memory task that required maintenance of the spatial positions of visual stimuli. Likewise, sleep-deprived subjects are impaired on tasks that require them to bind color-location pairings in working memory (Wee et al., 2013).

A working memory issue that has received more attention in the sleep literature than in research on amnesia is working memory updating (*cf.*, Spiers et al., 2001; Choo et al., 2005; Lythe et al., 2012). In many kinds of complex tasks, information must be continuously moved into and out of the focus of attention, and this process is often studied in the laboratory using the N-back task (Jonides et al., 1997; Pelegrina et al., 2015). Subjects performing the N-back task see a series of stimuli, often letters, and must report whether the stimulus on the current trial is the same as one N (typically from 1 to 3 items) back. Sleep-deprived subjects are impaired on the N-back task compared to their rested state (Choo et al., 2005). Relevant to the TASL framework is evidence that hippocampal-PFC pathways are invoked in the N-back task, which is consistent with the known role of the hippocampus in maintaining temporal order information (Costers et al., 2020). However, it is misleading to think of the N-back task as a simple measure of working memory updating because multiple cognitive processes are needed for successful performance including inhibition of distractors, and multiple interactions among the PFC, hippocampus, and basal ganglia are involved (Rac-Lubashevsky and Kessler, 2016; Costers et al., 2020). Hence, there are several potential sources of performance decrements on the N-back. More research is needed to determine whether the effects of sleep deprivation on the N-back are based on compromised hippocampal functioning, or due to other contributors to task performance.

Of course, sleep deprivation effects on cognition have often been attributed to deficits in PFC functioning and the circuits connecting the PFC to striatal and parietal areas (Chee et al., 2010; Wickens et al., 2015; Krause et al., 2017). The data on when STM and working memory tasks are, and are not, compromised by amnesia suggest that the need for binding in working memory tasks may well predict the degree of performance impairment from sleep deprivation, and serve as a further test of the TASL framework.

### 3.3. Binding in other cognitive domains

Studies of the effect of sleep deprivation on logical reasoning and complex decision-making have typically concluded that sleep deprivation has minimal effects on these cognitive processes (Harrison and Horne, 2000b; Killgore, 2010; Lim and Dinges, 2010). Similarly, preserved performance in these domains by people with amnesia was an important reason that amnesia was considered to be a specific deficit in declarative memory. But as we noted earlier, decision-making tasks such as the IGT, that require binding novel associations based on decision outcomes, are disrupted by MTL damage. A similar pattern of effects is observed with sleep deprivation during the IGT. Sleep-deprived subjects are less able to learn to choose from the advantageous decks on the IGT, resulting in poorer decision-making performance compared to controls (Killgore et al., 2006; Gupta et al., 2009).

The use of choice outcome feedback to guide future actions is a key component of adaptive behavior in everyday life, and it can mean the difference between success and disaster when making decisions in high stakes medical, military, and first responder settings. Consistent with the TASL framework, difficulty with binding choices and outcomes to guide future actions is not limited to the IGT. In amnesia patients and people deprived of sleep, impaired use of choice outcome feedback to guide subsequent choices has been demonstrated in decisions involving risk and in tasks requiring cognitive flexibility (Brand et al., 2009; Foerde et al., 2013; Whitney et al., 2015; Honn et al., 2019).

## 4. Conclusions and implications

Based on the accumulating evidence that the functioning of the hippocampus and associated MTL areas are disrupted by sleep deprivation in humans and in animal models (Tudor et al., 2016; Guttensen et al., 2023), it would be surprising if we did not observe significant effects of sleep loss on memory. However, to understand what aspects of memory are, and are not, affected by sleep deprivation requires a deeper understanding of the role of the hippocampus in memory. The TASL framework draws on the amnesia literature and the role of the hippocampus in binding to provide an organizing perspective on sleep deprivation and memory, and on how sleep loss is likely to affect other cognitive tasks that depend on binding.

The existing literature on sleep deprivation deficits in memory provides substantial support for the TASL framework. Although sleep deprivation does not lead to the same degree of impairment typically seen in amnesia resulting from MTL lesions, the pattern of impaired and preserved memory functioning observed in sleep-deprived subjects is remarkably consistent with that characteristically seen in amnesia patients. This is true not only for explicit recall and recognition tasks, but also for relational memory of items with their context. Even in the case of implicit memory, the pattern of preserved functions and deficits appears similar in amnesic patients and people who have had a night of sleep deprivation.

The TASL framework also calls attention to the possibility that some sleep deprivation effects outside of LTM may result from problems with relational binding. Amnesia patients show preserved STM maintenance along with deficits in working memory tasks that require processing of temporal, spatial, and other relational information, and this pattern has been observed after sleep loss as well.

The similarities between the effects of sleep deprivation and amnesia on various memorial processes lead to specific predictions that would test this framework. To begin, there remain several aspects of memory that have yet to be extensively or explicitly investigated under sleep deprivation, such as dissociating between explicit and implicit memories, or dissociating between tests that differ in their need for hippocampal involvement. From the TASL framework, we expect that sleep-deprived subjects would show similar patterns of performance as amnesia patients, insofar as the task is largely dependent on the hippocampus. Such a deliberate comparison would not only test the framework but would also provide clarity on how sleep deprivation and amnesia effects on memory differ. Separately, given that amnesia is characterized as a deficit in relational memory (Ryan et al., 2000), the TASL framework implies that sleep deprivation should result in impairment on all tasks that require binding, including those that do not fall under the memory domain. Such a prediction has wide-reaching implications with the obvious importance of binding to the development of schemas, inferences, and other forms of abstract representations that support flexible decision-making (Biderman et al., 2020; Vaidya and Badre, 2022). Further, because sleep deprivation does not result in deficits to the same extent as amnesia, we expect that any binding that does occur should result in less precise representations, as these similarly depend on intact hippocampal functioning (Ekstrom and Yonelinas, 2020). On a positive note, the TASL framework implies that some of the strategies that promote memory and performance in amnesia patients should also benefit sleep-deprived individuals. For example, unitizing or integrating separate representations into a single unit (e.g., creating the novel compound word “cloudlawn” to remember the words “cloud” and “lawn” together) has been shown to improve relational memory for certain types of amnesia (Quamme et al., 2007; Ryan et al., 2013). Testing these predictions will determine the extent to which the TASL framework is useful for guiding future research on sleep deprivation and memory-related processes.

One of the most important implications of the TASL framework is that while considerable research has documented sleep deprivation effects on LTM, disruptions in hippocampal functioning are expected to have specific consequences for relational processing needed on shorter timescales such as working memory processing, and on some kinds of decision-making tasks. In addition, the data we have reviewed here strongly suggest that to understand dissociations in the effects of sleep deprivation on cognitive performance, it will be important to not only examine what cognitive processes are needed for a given task, but also the *representations* on which the processes operate. The hippocampus and PFC-hippocampus interactions support multiple cognitive operations, but the contribution of this circuitry depends in part on the nature of the information being represented (Rubin et al., 2017). For example, as noted earlier, working memory is typically thought of as PFC driven processing, but the hippocampus is crucial when the information in the focus of attentional depends on maintenance of information about temporal or spatial relationships among elements.

Clearly, sleep loss disrupts more than the cognitive operation of binding, so the TASL framework can only serve as a guide to one source of impairment in cognitive performance. Nevertheless, future studies of the effects of sleep deprivation that examine whether it produces similar deficits, and islands of preserved performance, as found in the performance of amnesia patients on implicit memory, working memory, and decision-making, could help isolate the role of hippocampal disruption apart from other consequences of sleep loss.

## Author contributions

PW and JH contributed to the conception of the review. PW organized the structure of the manuscript. PW and CK wrote the original draft of the manuscript. All authors contributed to the article and approved the submitted version.

## Funding

This review was supported by National Institutes of Health grant R21CA167691 (JH) and Congressionally Directed Medical Research Program grant W81XWH-20-1-0442 (HVD).

## Conflict of interest

The authors declare that this research was conducted in the absence of any commercial or financial relationships that could be construed as a potential conflict of interest.

## Publisher’s note

All claims expressed in this article are solely those of the authors and do not necessarily represent those of their affiliated organizations, or those of the publisher, the editors and the reviewers. Any product that may be evaluated in this article, or claim that may be made by its manufacturer, is not guaranteed or endorsed by the publisher.
